# A Report of Some Surgical Cases*Read before Georgia Medical Association.

**Published:** 1898-10

**Authors:** T. R. Garlington

**Affiliations:** Rome, Ga.; Vice-President of the Association of Surgeons of the Southern Railway Company, Member of American Medical Association, Georgia Medical Association, Chief Surgeon C., R. & S. Ry. Co., Local Surgeon Southern Ry. Co., Surgeon City Electric Ry. Co.


### A REPORT OF SOME SURGICAL CASES.*

By T. R. GARLINGTON, M.D.,

Vice-President of the Association of Surgeons of the Southern Railway Company, Member of American Medical Association, Georgia Medical Association, Chief Surgeon C., R. & S. Ry. Co., Local Surgeon Southern Ry. Co., Surgeon City Electric Ry. Co.

The cases which I wish to report are deemed of sufficient interest to bring to the attention of the members of this Association.

The first case is one of empyema in an infant, aged seventeen months.

* Read before Georgia Medical Association.

The history of this case, as related by its mother, is to some extent interesting.

About 1st of February child caught cold, which was subsequently followed in three or four days by symptoms of earache, developing suddenly at night, and attended apparently by high temperature. After a while the child was soothed to sleep, and the next morning she still had fever. The family physician was called and made a diagnosis of pneumonia, for which she was treated for three or four weeks; and as her condition continued to grow worse a consultation was asked for, which was had. A diagnosis of unresolved pneumonia was agreed upon.

Vigorous treatment was instituted to induce resolution; however, all measures of course failed. The child's condition grew so desperate that her physicians despaired of her recovery, and so informed the parents.

I was then called, and made my first visit on April 28th; the child had been ill about twelve weeks.

The symptoms were extreme pallor with profound emaciation; respiration very greatly embarrassed; a continuous hacking cough; pulse weak and greatly accelerated; chest bulging or barrel-shaped on right side, with marked dullness on percussion; auscultation negative; left side retracted and slightly resonant; weak vesicular breathing.

I made a diagnosis of empyema, which was confirmed by aspiration. I advised an immediate operation, which was consented to; but as the child's condition was so extremely critical I could not make a favorable prognosis.

I made an incision in the sixth interspace, and drained the cavity with rubber drainage-tubes.

I am quite sure fully half a gallon of pus was discharged from time to time.

Irrigation was not resorted to, as I consider it to some extent dangerous.

I removed the tubes in about two weeks, and my little patient made a complete recovery.

RECAPITULATION.

There are two points worthy of notice in this case: First, the age of the patient at which this condition developed, and, second, the fact that an error was made in the diagnosis.

That pleurisy with effusion and empyema is quite a common complication with pneumonia in infancy and childhood there can be no doubt. And that errors in diagnosis are commonly made is evidenced by the above case, together with two others which I have quite recently operated on, in which errors were made in both cases. In one, a diagnosis of spinal curvature was made and a plaster jacket was about to be applied. This common error can be avoided if the physician will take the pains to make a careful physical examination of the patient's chest; and should any doubt exist, aspiration with a hypodermic needle should be made, which would confirm a diagnosis of pleurisy with effusion or an empyema.

CASE NO. 2.—This patient, Jesse H. White, aged about twenty-two, was an employee of the Southern Railway Company and a member of the bridge gang.

While repairing a trestle he lost his footing and was precipitated to the ground, a distance of about twenty-five or thirty feet. He fell across a mudsill at the bottom of an excavation, producing a compound comminuted fracture of the femur at the junction of the upper and middle third, with great laceration and contusion of subcutaneous tissue.

A physician living in the country near-by was called, and he applied a temporary dressing. Four days later I was called to the patient, and upon my arrival I found him with acute septicemia complicating his injury, with a temperature of 105.

I removed the bandages and splints, and a great deal of foul pus was discharged.

Ecchymosis was very pronounced and extended from the knee to a point just above the iliac crest on the abdomen.

I recognized at once that I had a very grave condition to deal with to save my patient's life, to say nothing of his leg.

I enlarged the wound and made a counter incision about six inches long down to the bone on the back of the thigh, and after thorough irrigation with 1-2000 bichloride solution I drained with large gauze ropes, and then placed my patient on quinine and whisky. In about a week his temperature came to normal, and a week later I performed a resection.

When I made my incision I found the bone denuded of its periosteum and apparently dead for several inches on both the lower and upper fragments. I resected about two and a half inches from the lower and about two inches from the upper fragment; in neither instance did I get beyond the denuded surface. After resecting about one inch from upper fragment I found the medullary canal suppurating and discharging very foul pus. I went an inch higher, endeavoring to get beyond the point of suppuration, which, however, I failed to do.

As my patient was taking ether badly I concluded to stop at this and take chances on the result.

I drilled and wired the bones end to end with a large silver wire, leaving the ends long and bringing them out through the incision. I closed the wound both above and below and drained through and through with large rubber drainage-tubes. I used for dressing the wound four cardboard splints molded to fit the thigh, and then applied sandbags.

After three or four days I substituted a plaster-cast, with fenestra. The wound was irrigated with bichloride solution 1-2000 and dressed every second or third day for two weeks, at which time the wires were removed; aftewards dressed as the occasion demanded.

I allowed the plaster-cast to remain on about four weeks, and after its removal I applied sandbags and kept my patient in bed four to six weeks longer.

I obtained a most excellent result, and my patient is to-day well and hearty, and is employed as fireman at the Massachusetts Cotton Mills in Georgia, where he informs me that he lifts several tons of coal every day, and has as good a leg as anybody.

SUMMARY.

This case serves to illustrate well the reparative properties of bone, and I herewith make a plea for conservatism in all railway as well as other injuries to bone, and especially among the laboring classes.

